# Supplementary material for: Massive carbon storage in convergent margins initiated by subduction of limestone
Source: Nat Commun. 2021 Jul 22;12:4463. doi: 10.1038/s41467-021-24750-0 (PMC8298627; doi:10.1038/s41467-021-24750-0)
Supplement: Supplementary file 1 — Supplementary Information [file 41467_2021_24750_MOESM1_ESM.pdf]

Supplementary information

Massive carbon storage in convergent margins initiated by subduction of limestone

Chunfei Chen, Michael W. Förster, Stephen F. Foley, Yongsheng Liu

Data sources for Fig. 1

Global limestones from Frimmel[1], Qiu, et al.[2], Thomas and Aitchison[3], Tsikos, et al.[4], Abdel-Rahman and Nader[5], and Armstrong-Altrin, et al.[6]. The chalks from Farouk, et al.[7].

Arc peridotites including harzburgites and dunites from Pearce, et al.[8], Ionov[9]. Czertowicz, et al.[10], and Marchesi, et al.[11].

Supplementary Fig. 1:

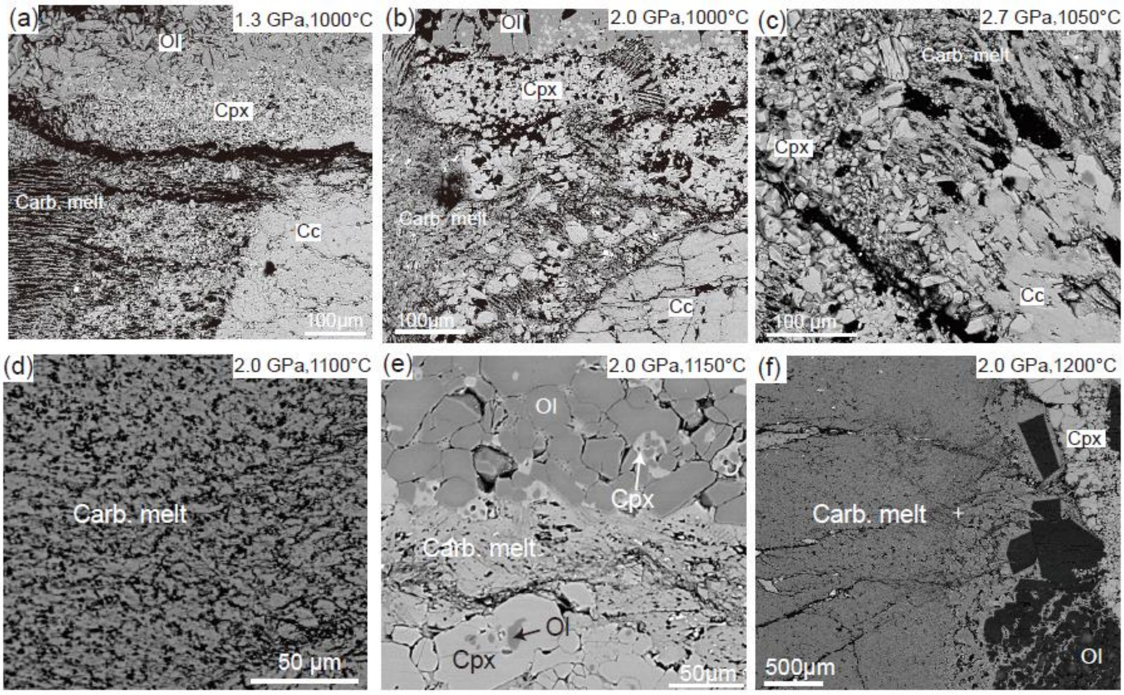

Supplementary Fig. 1. Backscattered electron images of carbonatite melts in the reaction zones between limestone and dunite layers in the experiments performed at (a) 1.3 GPa, (b) 2.0 GPa, and (c) 2.7 GPa. Near-solidus carbonatite melts occur in the reaction zone dominated by clinopyroxene. (d) shows the structure of the carbonatite melt without quenched minerals. Relict olivine inclusions overgrown by newly grown clinopyroxenes show that the clinopyroxene layer replaces the dunite (e). (f) The limestone layer is entirely molten at 1200 °C and 2.0 GPa. The dunite layer was removed to the right of the capsule during melting.

Supplementary Fig. 2:

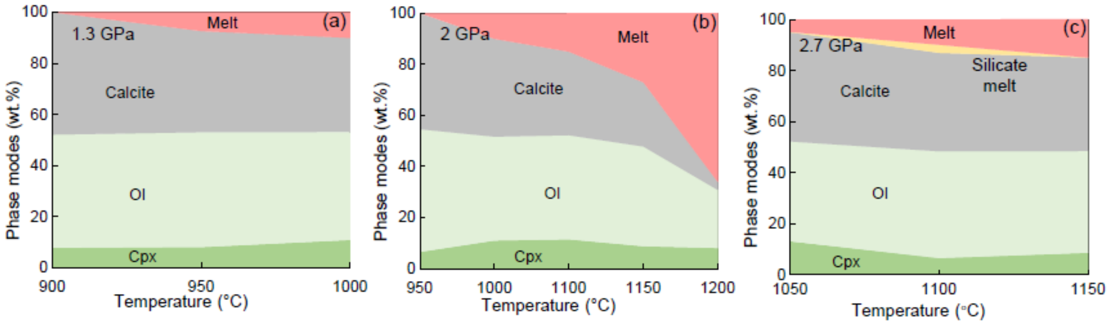

Supplementary Fig. 2: The variation of mineral and melt modes with increasing temperature at various pressures (Supplementary Table 1). Ol-olivine, Cpx-clinopyroxene, Carb.melt-carbonatite melt, and Sili.melt-Silicate melt. Compared to the experiments of 1050 °C and 1150 °C at 2.7 GPa, lower Cpx mode at 1100 °C and 2.7 GPa is attributed to no complete reaction between silicate melt and olivine (and calcite) in the capsule. Note: the temperatures on the x-axis are not evenly spaced.

Supplementary Fig. 3:

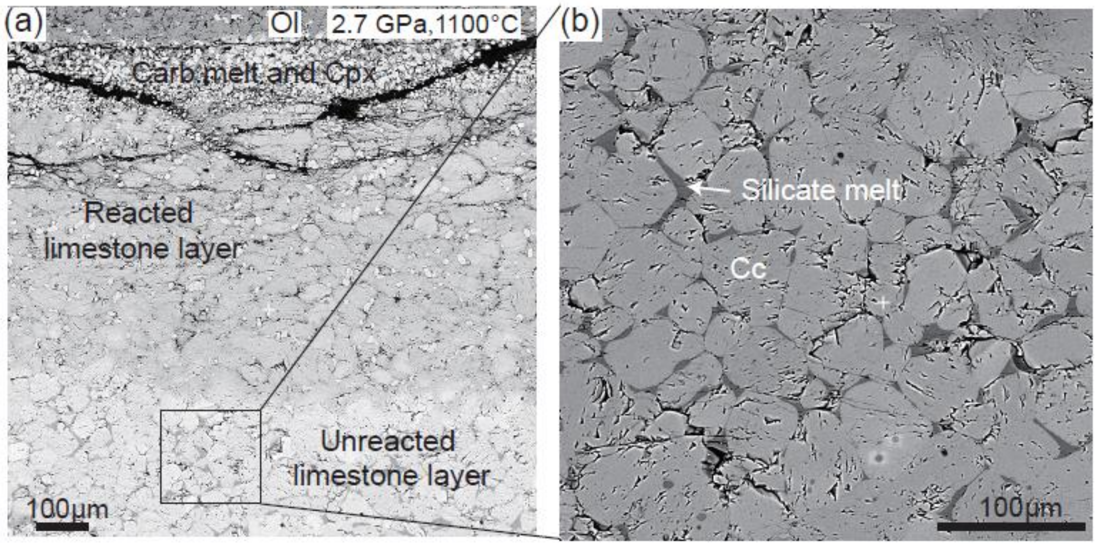

Supplementary Fig. 3: Backscattered electron images of experimental products at 2.7 GPa and 1100 °C. Silicate melts occur along boundaries between calcite crystals in the former limestone zone that has not reacted with peridotite.

Supplementary Fig. 4:

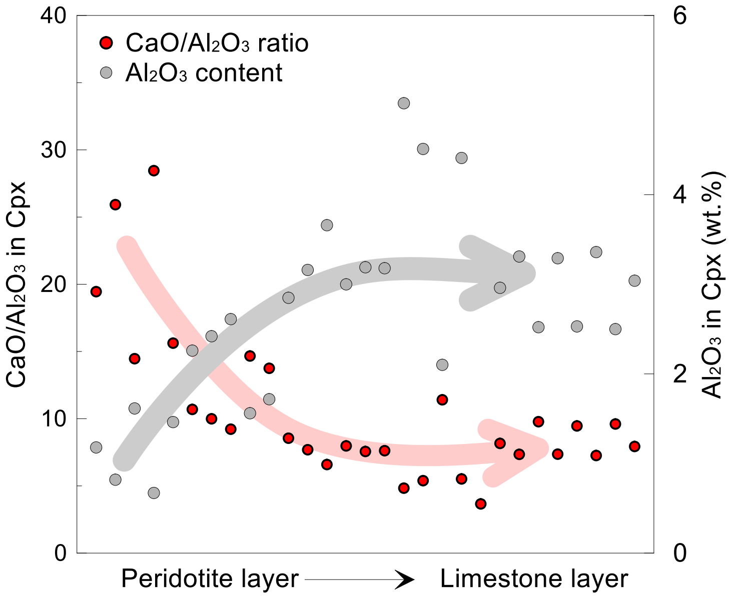

Supplementary Fig. 4: The variation of CaO/Al_2O_3 ratio (pink) and Al_2O_3 content (grey) in newly grown clinopyroxenes across the capsule from the peridotite layer to the limestone layer (experiments at 2 GPa, 1150 °C). The CaO/Al_2O_3 ratio and Al_2O_3 contents decrease and increase from the peridotite layer to the limestone layer, respectively.

Supplementary Fig. 5:

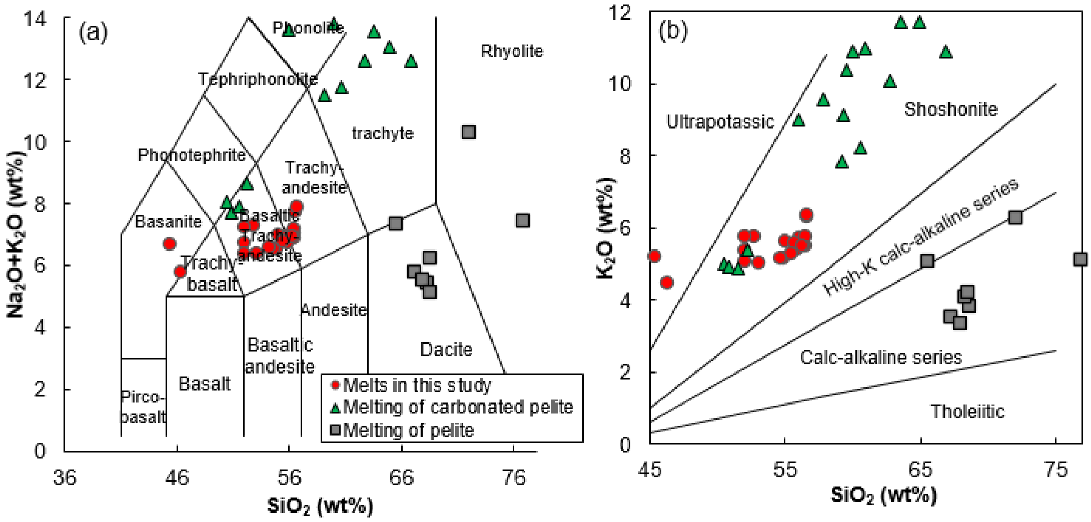

Supplementary Fig. 5: Major element compositions of silicate melts in the experiment at 2.7 GPa and 1100 °C compared to experimental silicate melts from melting of carbonated pelite[12] and pelite without carbonate[13].

Supplementary Fig. 6:

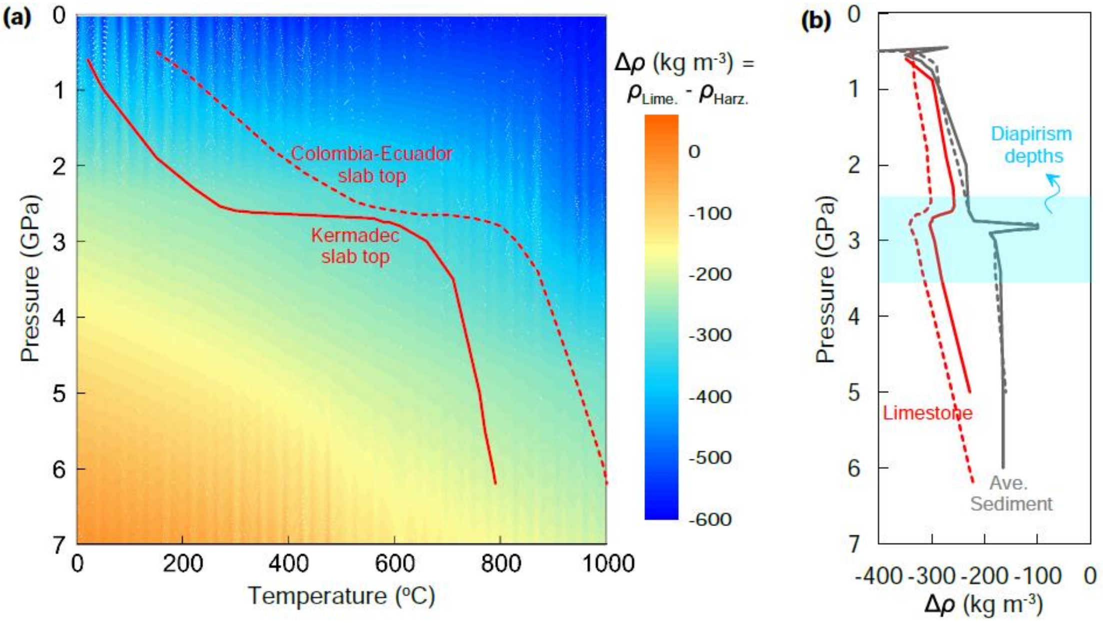

Supplementary Fig. 6: Calculated density of limestone (starting material used in our high-pressure experiment) along typical subduction zone geotherms using Perple_X 6.9.0[14]. (a) Density contrast between the limestone and mantle wedge harzburgite[15] as a function of temperature and pressure. Red solid and dotted lines represent slab-top geotherms for Kermadec and Colombia-Ecuador[16], respectively. (b) Density contrast as a function of pressure along Kermadec (red solid line) and Colombia-Ecuador (red dotted line) slab geotherms. The density contrast of the average ultrahigh-pressure metasediment along Izu-Bonin (cold subduction, grey solid line) and Cascadia (warm subduction, grey dotted line) from Behn, et al.[17] are shown for comparison. The

calculated depths of diapir initiation for sediments involving linestone (2.4-3.5 GPa, see main text) are shown.

Supplementary Fig. 7:

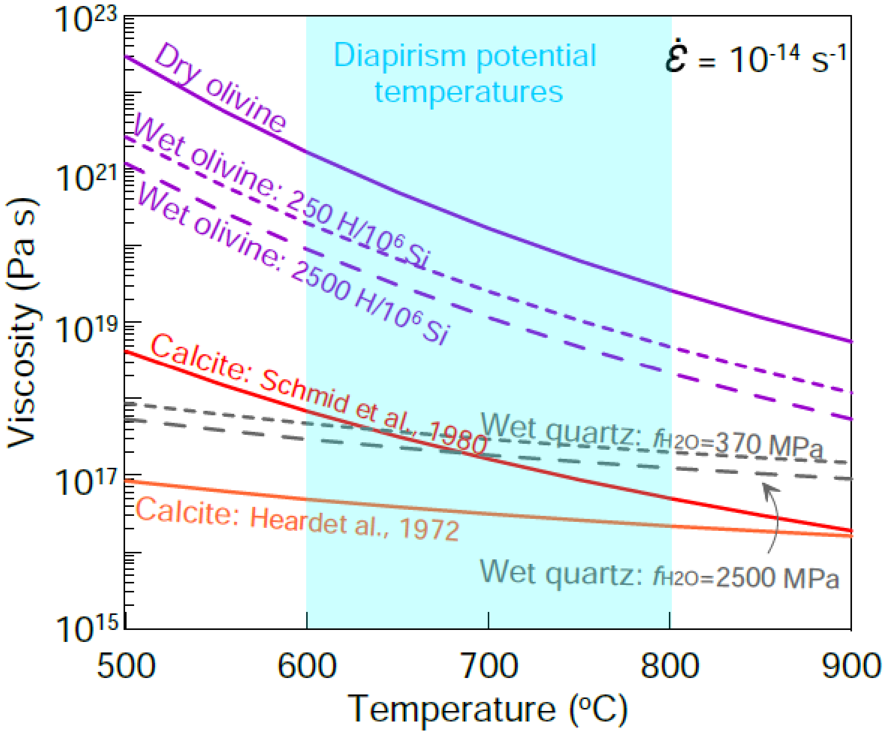

Supplementary Fig. 7: Flow viscosity of dry and wet olivines (C_{OH} = 250 and 2500 H/10^6Si)[18], wet quartz (f_{H2O} = 370 and 2500 MPa)[19], and calcite[20,21] as a function of temperature at a strain rate of 10^{-14} s^{-1}. Note that calcite from Schmid, et al.[20] has a similar or slightly lower viscosity than wet quartz, but about 100 × less viscous than that of wet olivine at the temperature of 600-800 °C (the approximate temperature range over which diapirs form).

Supplementary Fig. 8:

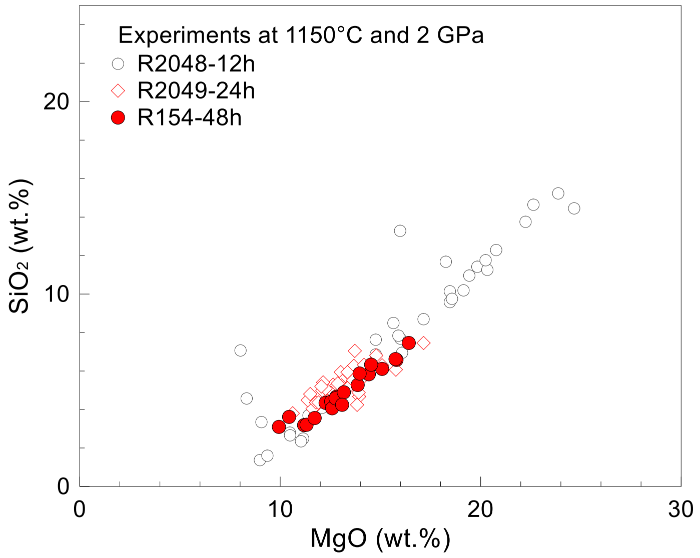

Supplementary Fig. 8: Effect of experimental duration (12 h, 24 h, and 48 h) on MgO and SiO_2 contents of carbonatite melts at 1150 °C and 2 GPa. Carbonatite melts for 24 h and 48 h experiments are more homogeneous than those of the 12 h experiment, indicating that equilibrium is approached after 24 hours.

Supplementary Table 1 The conditions and results of the reaction experiments between limestone and dunite.

NO.	P (GPa)	T (°C)	Duration (h)	Mineral modes (wt.%)						∑R^2	Fe loss (wt.%)
				Ol	Calcite	Mg-calcite	Cpx	Carb. Melt	Sil. Melt		
R162	1.3	900	120	48	45	2	5			1.48	12.3
R166	1.3	950	120	45	39		8	9		0.46	8.5
R165	1.3	1000	108	42	37		11	10		0.73	11.3
R163	2.0	950	120	47	46	2	5			1.24	3.9
R2063	2.0	1000	120	42	43		11	5		0.01	9.5
R157	2.0	1100	48	41	33		11	15		0.49	14.6
R2048	2.0	1150	12	39	32		10	19		0.88	19.2
R2049	2.0	1150	24	40	31		10	19		0.85	33.6
R154	2.0	1150	48	37	30		13	20		1.08	31.6
R150	2.0	1200	48	26	0		6	68		2.68	63.7
MO-20-28	2.7	1000	60	48	42	4	5			0.46	3.8
CH-3	2.7	1050	24	39	43		13	5		0.54	23.7
CH-1	2.7	1100	24	44	27	10	5	11	3	0.13	17.1
CH-2	2.7	1150	24	34	38		12	16		0.52	26.5

All experiments are reaction experiments between limestone and dunite by packing limestone and dunite powders with a 1:1 weight ratio as separate blocks in the capsule. The phase modes were estimated by mass balance calculation which is presented in the Methods and materials in detail. Note: Ol-olivine, Cc-Calcite, Cpx-Clinopyroxene, Carb.melt-Carbonatite melt, Sil.melt-Silicate melt.

Supplementary Table 2. Major element compositions of the starting materials and averages of carbonatite melt and silicate melt in the experiments.

NO.	Comment	P GPa	T °C	Time h	n	SiO_2	TiO_2	Al_2O_3	FeO	MnO	MgO	CaO	Na_2O	K_2O	Na_2O+K_2O	SO_2	H_2O	Total	Ca/(Ca+Mg) atomic ratio
Limestone	SM WR					4.5	0.06	0.81	0.32	0.03	0.6	50.3	0.3	0.01		0.24	0.90	56.7	
	SM Clay (7.4%)					61.3	0.75	10.9	4.3	0.42	2.71		4.11	0.10		3.31	12.1	100.0	
Dunite	SM					41.1	0.03	0.02	9.98	0.14	48.2	0.03	0.01	0.01				99.5	
R166	Carbonatite melt	1.3	950	120	6	4.09	0.07	0.85	3.47	0.07	6.14	37.2	0.40	0.18	0.58			52.5	0.81
					sd	1.19	0.05	0.42	1.61	0.07	1.4	6.66	0.18	0.09					
R165	Carbonatite melt	1.3	1000	108	10	2.56	0.08	0.29	3.17	0.07	7.44	34.8	0.28	0.15	0.43			48.8	0.77
					sd	0.59	0.06	0.13	0.67	0.07	1.48	2.87	0.09	0.05					
R2063	Carbonatite melt	2	1000	120	5	3.31	0.09	0.76	4.43	0.12	9.39	33.2	0.43	0.17	0.60			51.8	0.72
					sd	0.63	0.06	0.27	0.86	0.03	3.85	0.69	0.4	0.08					
R157	Carbonatite melt	2	1100	48	9	2.97	0.09	0.61	4.42	0.12	10.5	32.2	0.62	0.41	1.03			51.9	0.69
					sd	2.43	0.08	0.53	2.34	0.02	2.9	5.86	0.42	0.5					
R154	Carbonatite melt	2	1150	48	20	4.9	0.04	0.45	2.5	0.11	13.2	37.3	0.19	0.15	0.34			58.8	0.67
					sd	1.3	0.02	0.2	0.45	0.02	1.8	1.46	0.18	0.09					
R150	Carbonatite melt	2	1200	24	30	13.3	0.03	0.64	1.2	0.07	15.7	36.7	0.12	0.06	0.18			67.8	0.63
					sd	1.18	0.02	0.18	0.11	0.02	1.58	1.38	0.05	0.03					
R151	Carbonatite melt	2	1250	24	50	10.7	0.04	0.83	2.03	0.08	14.2	35.8	0.23	0.12	0.35			64.0	0.64
					sd	1.29	0.02	0.24	0.21	0.02	1.18	1.09	0.08	0.05					
CH3	Carbonatite melt	2.7	1050	24	13	4.12	0.05	1.18	3.28	0.13	13.2	35.5	0.27	0.4	0.67			58.2	0.66
					sd	1.57	0.04	0.75	0.96	0.05	2.06	2.08	0.07	0.27					
CH1	Carbonatite melt	2.7	1100	24	5	2.5	0.05	0.08	1.89	0.1	11.1	41.2	0.15	0.07	0.22			66.1	0.73
					sd	3.09	0.05	0.03	0.55	0.02	2.86	3.1	0.09	0.04					
	Silicate melt				18	54.9	0.69	15.4	1.64	0.17	1.27	8.64	1.40	5.57	6.97			89.73	
					sd	1.7	0.1	0.7	0.2	0.0	0.3	1.1	0.1	0.4					
CH2	Carbonatite melt	2.7	1150	24	9	5.25	0.1	0.36	2.92	0.07	15.6	35.7	0.15	0.08	0.23			60.3	0.62
					sd	1.51	0.07	0.23	0.93	0.05	1.86	2.34	0.12	0.07					

Note: The compositions of melts are averages of melts in the experiment. The detailed data are given in Supplementary Data 1.

Supplementary references:

1. Frimmel, H. E. Trace element distribution in Neoproterozoic carbonates as palaeoenvironmental indicator. *Chemical Geology* **258**, 338-353 (2009).

2. Qiu, Z., Wang, Q. & Yan, D. Geochemistry of the Middle to Late Permian limestones from the marginal zone of an isolated platform (Laibin, South China). *Sci. China Earth Sci.* **56**, 1688-1700 (2013).

3. Thomas, C. W. & Aitchison, J. Log-ratios and geochemical discrimination of Scottish Dalradian limestones: a case study. *Geological Society, London, Special Publications* **264**, 25-41 (2006).

4. Tsikos, H., Moore, J. M. & Harris, C. Geochemistry of the Palæoproterozoic Mooidraai Formation: Fe-rich limestone as end member of iron formation deposition, Kalahari Manganese Field, Transvaal Supergroup, South Africa. *Journal of African Earth Sciences* **32**, 19-27 (2001).

5. Abdel-Rahman, A. F. M. & Nader, F. H. Characterization of the Lebanese Jurassic–Cretaceous carbonate stratigraphic sequence: a geochemical approach. *Geological Journal* **37**, 69-91 (2002).

6. Armstrong-Altrin, J. S., Verma, S. P., Madhavaraju, J., Lee, Y. I. & Ramasamy, S. Geochemistry of Upper Miocene Kudankulam Limestones, Southern India. *International Geology Review* **45**, 16-26 (2003).

7. Farouk, S. *et al.* Geochemical characteristics of carbonaceous chalk near the Cretaceous/Paleogene transition, central Jordan: Strong metal enrichment of redox-sensitive and biophile elements from remineralized calcitic plankton. *Marine and Petroleum Geology* **120**, 104535 (2020).

8. Pearce, J. A., Barker, P. F., Edwards, S. J., Parkinson, I. J. & Leat, P. T. Geochemistry and tectonic significance of peridotites from the South Sandwich arc-basin system, South Atlantic. *Contrib Mineral Petrol* **139**, 36-53 (2000).

9. Ionov, D. A. Petrology of Mantle Wedge Lithosphere: New Data on Supra-Subduction Zone Peridotite Xenoliths from the Andesitic Avacha Volcano, Kamchatka. *Journal of Petrology* (2010).

10. Czertowicz, T. A. *et al.* The Anita Peridotite, New Zealand: Ultra-depletion and Subtle Enrichment in Sub-arc Mantle. *Journal of Petrology* **57**, 717-750 (2016).

11. Marchesi, C. *et al.* Geochemical record of subduction initiation in the sub-arc mantle: Insights from the Loma Caribe peridotite (Dominican Republic). *Lithos* **252-253**, 1-15 (2016).

12. Thomsen, T. B. & Schmidt, M. W. Melting of carbonated pelites at 2.5–5.0 GPa, silicate–carbonatite liquid immiscibility, and potassium–carbon metasomatism of the mantle. *Earth and Planetary Science Letters* **267**, 17-31 (2008).

13. Wang, Y., Foley, S. F. & Prelević, D. Potassium-rich magmatism from a phlogopite-free source. *Geology* **45**, 467-470 (2017).

14. Connolly, J. A. D. Computation of phase equilibria by linear programming: A tool for geodynamic modeling and its application to subduction zone decarbonation. *Earth and Planetary Science Letters* **236**, 524-541 (2005).

15. Jull, M. & Kelemen, P. On the conditions for lower crustal convective instability. *JOURNAL OF GEOPHYSICAL RESEARCH* **106**, 6423-6446 (2001).

16. Syracuse, E. M., van Keken, P. E. & Abers, G. A. The global range of subduction zone thermal models. *Physics of the Earth and Planetary Interiors* **183**, 73-90 (2010).

17. Behn, M. D., Kelemen, P. B., Hirth, G., Hacker, B. R. & Massonne, H.-J. Diapirs as the source of the sediment signature in arc lavas. *Nature Geoscience* **4**, 641-646 (2011).

18. Hirth, G. & Kohlstedf, D. Rheology of the upper mantle and the mantle wedge: A view from the experimentalists. *Geophysical Monograph-American Geophysical Union* **138**, 83-106 (2003).

19. Hirth, G., Teyssier, C. & Dunlap, J. W. An evaluation of quartzite flow laws based on comparisons between experimentally and naturally deformed rocks. *International Journal of Earth Sciences* **90**, 77-87 (2001).

20. Schmid, S. M., Paterson, M. S. & Boland, J. N. High temperature flow and dynamic recrystallization in carrara marble. *Tectonophysics* **65**, 245-280 (1980).

21. Heard, H. C. & Raleigh, C. B. Steady-State Flow in Marble at 500° to 800°C. *GSA Bulletin* **83**, 935-956 (1972).
