# Supplementary material for: Massive carbon storage in convergent margins initiated by subduction of limestone
Source: Nat Commun. 2021 Jul 22;12:4463. doi: 10.1038/s41467-021-24750-0 (PMC8298627; doi:10.1038/s41467-021-24750-0)
Supplement: Supplementary file 2 — Description of Additional Supplementary Files [file 41467_2021_24750_MOESM2_ESM.docx]

**Description of Additional Supplementary File**

Supplementary Data 1.

Major element compositions of silicate melts, carbonatite melts, and minerals in the experiments.
